# A randomized, double-blind, placebo-controlled, crossover study to evaluate the human abuse liability of solriamfetol, a selective dopamine and norepinephrine reuptake inhibitor

**DOI:** 10.1177/0269881118796814

**Published:** 2018-10-01

**Authors:** Lawrence P Carter, Jack E Henningfield, Y Grace Wang, Yuan Lu, Debra Kelsh, Bradley Vince, Edward Sellers

**Affiliations:** 1 Jazz Pharmaceuticals, Palo Alto, CA, USA; 2 Department of Pharmacology & Toxicology, University of Arkansas for Medical Sciences, Little Rock, AR, USA; 3 Pinney Associates, Bethesda, MD, USA; 4 Department of Psychiatry and Behavioral Sciences, Johns Hopkins School of Medicine, Baltimore, MD, USA; 5 Vince & Associates Clinical Research, Overland Park, KS, USA; 6 Department of Pharmacology and Toxicology, Medicine, and Psychiatry, University of Toronto, ON, Canada

**Keywords:** Human abuse potential, solriamfetol, JZP-110, DNRI

## Abstract

**Background:**

This study evaluated the human abuse potential of solriamfetol (formerly JZP-110), a selective dopamine and norepinephrine reuptake inhibitor with robust wake-promoting effects.

**Methods:**

Adults with a recent history of recreational polydrug use, including stimulants, and who met criteria in a Qualification Phase were randomized to one of six sequences in a Test Phase. Each Test Phase sequence included a single administration of placebo, solriamfetol (300, 600, and 1200 mg), and phentermine (45 and 90 mg), with a two-day washout between periods. The primary endpoint was peak rating (*E*_max_) of Liking at the Moment across the first 12 h on a liking/disliking visual analog scale; key secondary endpoints were Next Day Overall Drug Liking, how much the participant would like to Take the Drug Again, and positive and negative subjective effects. Safety was also assessed throughout the study.

**Results:**

Of 43 participants (74.4% male; mean age 29.3 years), 37 completed the study. Peak *E*_max_ Liking at the Moment for all solriamfetol doses was significantly greater than placebo and significantly less than phentermine 90 mg (*p* < 0.05). Overall Next Day Drug Liking was greater than placebo for solriamfetol 300 mg and phentermine 45 and 90 mg (*p* < 0.05). Willingness to Take the Drug Again was significantly greater than placebo and significantly less than both doses of phentermine for all doses of solriamfetol (*p* < 0.05). Ratings of negative subjective effects (bad effects, disliking, anxiety, agitation) were higher with solriamfetol 600 and 1200 mg relative to phentermine. The most common treatment-emergent adverse events with solriamfetol were hypervigilance, elevated mood, dry mouth, hyperhidrosis, and insomnia.

**Conclusion:**

Solriamfetol appears to have abuse potential similar to or lower than phentermine.

## Introduction

Excessive sleepiness (hypersomnolence) is a prominent characteristic of a variety of medical and psychiatric conditions that include disorders of central hypersomnia (e.g. narcolepsy), sleep-related breathing disorders (e.g. obstructive sleep apnea; OSA), and neurodegenerative conditions (e.g. Parkinson’s disease) (American Academy of Sleep Medicine, 2014). Traditional stimulants (amphetamines, methylphenidate) and wake-promoting agents (modafinil, armodafinil) are used to treat excessive sleepiness in these conditions; however, these drugs are associated with limitations that include abuse potential, and poor tolerability and/or suboptimal response in some patients (Rosenberg, 2015, Takenoshita and Nishino, 2017; Thorpy and Dauvilliers, 2015). Consequently, there remains a need for additional therapeutic options for patients with excessive sleepiness, which has been shown to contribute to the substantial economic and humanistic burdens associated with these diseases. These burdens include increased healthcare resource utilization, reductions in quality of life and work productivity, and higher risk of motor vehicle and occupational accidents relative to the general population (Black et al., 2014; Flores et al., 2016; Garbarino et al., 2016; Hirsch Allen et al., 2015; Moyer et al., 2001; Philip et al., 2010).

Solriamfetol (JZP-110) is a selective dopamine and norepinephrine reuptake inhibitor (DNRI) with robust wake-promoting effects that is being developed to improve wakefulness and reduce excessive sleepiness associated with narcolepsy, OSA, and Parkinson’s disease. In 12-week clinical trials in adults with narcolepsy (Bogan et al., 2015; Ruoff et al., 2016), solriamfetol had robust effects at doses of 150 to 300 mg/day (the highest planned therapeutic dose) one week after the beginning of treatment, with significant reductions in participant-reported excessive sleepiness measured on the Epworth Sleepiness Scale (ESS), and global improvements assessed by participants and physicians compared with placebo. Objective assessment of the ability to stay awake on the Maintenance of Wakefulness Test (MWT) indicated significant improvements from baseline relative to placebo with both solriamfetol doses. In two randomized, double-blind, placebo-controlled studies in participants with OSA, solriamfetol also demonstrated significant improvements from baseline in ESS scores and MWT sleep latency at six weeks (Strohl et al., 2017) and at 12 weeks (Strollo et al., 2017).

Solriamfetol is a selective DNRI with effects that appear to be distinct from those of traditional stimulants. In vitro studies have shown that solriamfetol, at concentrations in the micromolar range, selectively bound to and inhibited reuptake at dopamine and norepinephrine transporters without promoting the release of monoamines (Baladi et al., 2018; Carter et al., 2016). Like other drugs that inhibit dopamine or dopamine and norepinephrine reuptake, such as modafinil and bupropion, respectively, the discriminative stimulus effects of solriamfetol generalized to a cocaine discriminative stimulus in rats and rhesus monkeys (Baladi et al., 2017, 2018). Also, consistent with the pharmacological profile of a reuptake inhibitor versus a monoamine releaser, the effects of solriamfetol on increasing locomotor behavior have been shown to be less than those of traditional stimulants (Baladi et al., 2018; Carter et al., 2016), and solriamfetol did not produce significant conditioned place preference or maintain intravenous self-administration in rats. Although these in vivo results suggest a low potential for abuse, it is important (and required) by the United States Food and Drug Administration (FDA) to provide a clinical assessment of human abuse liability (HAL) or human abuse potential to characterize the safety profile of a new medication such as solriamfetol. This type of assessment can inform healthcare decisions with regard to its abuse potential and scheduling. In addition, the assessment of the relative abuse potential of drugs with similar effects, but different pharmacological mechanisms of action, advances our general understanding of the relationship between the pharmacology and abuse potential of different drugs. Thus, this study was designed to evaluate the human abuse potential of solriamfetol relative to placebo and the Schedule IV stimulant phentermine as a positive control.

## Materials and methods

### Study design

This study received Institutional Review Board approval from Midlands Independent Review Board (Overland Park, KS, USA) and was conducted in accordance with Good Clinical Practice and the Declaration of Helsinki; all participants provided written informed consent. Design and implementation of the study was also consistent with the 2010 FDA draft guidance for HAL studies (US Food and Drug Administration and Center for Drug Evaluation and Research, 2010), which was finalized after this study concluded (US Food and Drug Administration and Center for Drug Evaluation and Research, 2017). The study used a randomized, double-blind, placebo-controlled, crossover design that included a Screening Phase, a Qualification Phase, and a Test Phase. The Qualification and Test Phases were conducted in a closed residential research unit where caffeine was not available and smoking was limited to certain times of the day. On dosing days, cigarette smokers were allowed to smoke one cigarette upon rising and then were not allowed to smoke until after the 6-h assessment was completed. Smoking was not allowed within 30 min prior to the eight- and 12-h assessments; smoking was allowed on washout and non-dosing days after vital signs and any scheduled 24-h assessments were completed.

Participants were evaluated for eligibility during the Screening Phase. A standard medical evaluation was conducted that included a medical history and physical examination, vital signs assessment, 12-lead electrocardiography, and laboratory evaluations.

Participants who met eligibility criteria entered a six-day Qualification Phase in which they were randomized (1:1) to receive either a sequence of placebo on day 1 and phentermine 60 mg on day 4 or a sequence of phentermine 60 mg on day 1 and placebo on day 4 under double-blind conditions. Participants who tolerated phentermine in the Qualification Phase and who reported greater liking for phentermine versus placebo, time-dependent effects, and neutral liking for placebo were enrolled in the Test Phase. Greater liking was defined as peak liking ⩾15 points higher for phentermine versus placebo on a 0–100 bipolar liking/disliking visual analog scale (VAS; 0 = strong disliking and 100 = strong liking) and neutral liking was a VAS score between 40 and 60.

Participants who met the Qualification Phase criteria were randomized in the Test Phase to one of six double-blind treatment sequences generated using the Williams method for Latin Square design. A statistician who had no contact with the participants nor involvement with assessment of their eligibility prepared and retained the master randomization code for both the Qualification and Test Phases. All study personnel except the study pharmacists were blinded to the study treatments, which were prepared in identical-looking capsules. The six treatments included single administration of placebo; solriamfetol 300, 600, and 1200 mg; and phentermine 45 and 90 mg. The FDA Guidance for the Assessment of the Abuse Potential of Drugs recommends that supratherapeutic doses two to three times the proposed therapeutic dose be assessed in HAL studies (US Food and Drug Administration and Center for Drug Evaluation and Research, 2017), and other expert reviews urge higher doses if they can be safely administered (Carter and Griffiths, 2009; Griffiths et al., 2003). Thus, choice of solriamfetol doses was based on results from previous clinical studies of solriamfetol in which 300 mg was the highest therapeutic dose (Bogan et al., 2015; Ruoff et al., 2016); 1200 mg is the highest dose that has been administered to human participants and was considered safe to administer in this study.

Comparisons of solriamfetol with both placebo and a positive control were performed, consistent with FDA guidance (US Food and Drug Administration and Center for Drug Evaluation and Research, 2017), to enable evaluation of acute effects of single-dose administrations of drugs over a period of time commensurate with the time course of the relevant drug effects. Phentermine was used as the positive control at the studied doses because it is a Schedule IV stimulant drug with previously established measurable abuse potential at these doses (Jasinski et al., 2008; Schoedel et al., 2012). Phentermine is a well-characterized amphetamine-type central nervous system stimulant that releases norepinephrine, and does so at a similar potency as amphetamine and methamphetamine (Rothman et al., 2001). There also is recognized epidemiologic potential for the abuse of non-amphetamine stimulants such as phentermine as indicated by substantial numbers of emergency department visits related to the use of such non-amphetamine anorexiants and stimulants (Substance Abuse and Mental Health Services Administration, 2013), and reports of intentional exposure to non-amphetamine diet aids and stimulants to Poison Control Centers (Mowry et al., 2013). Due to its robust stimulant effects and lower scheduling status, phentermine is a useful positive control for the evaluation of the human abuse potential of medications that have a pharmacological mechanism of action similar to that of traditional stimulants, but low suspected potential for abuse based on non-clinical assessments. Thus, phentermine was considered to represent the most appropriate pharmacological class (stimulants) and drug to which solriamfetol (reuptake inhibitor with low likely potential for abuse) should be compared.

Dosing days were separated by two days to allow for washout between experimental conditions. This washout period of 72 h is approximately four times the mean terminal *t*_1/2_ of 20 h for phentermine (VIVUS, 2014), and is greater than five times the *t*_1/2_ of solriamfetol (5–6 h) (Zomorodi et al., 2017).

### Participants

For inclusion, male and female participants were required to be 18–55 years old, inclusive, with a body mass index 18–32 kg/m^2^, inclusive, and have a self-reported history of recreational polydrug use from ⩾2 illicit drug classes including a stimulant (i.e. cocaine, amphetamine, methamphetamine, methylphenidate, or phentermine). Recreational stimulant use ⩾10 times in the past five years and at least once in the past three months was also required. Key exclusion criteria were therapeutic use of central nervous system-acting drugs or drugs that modulate monoaminergic signaling; monoamine oxidase inhibitors within two weeks of the Qualification Phase; daily caffeine use at the Screening Phase >600 mg/day of caffeine or >6 cups of coffee/day; daily cigarette use >20 cigarettes/day or any other daily use of nicotine-containing products; history or presence of any clinically significant or unstable medical condition, behavioral or psychiatric disorder or surgical history that could affect the safety of the participant or interfere with study assessments per the judgment of the investigator; positive screen for human immunodeficiency virus antibodies, hepatitis B virus antigens, hepatitis C virus antibodies, hepatitis A IgM antibodies, or a clinical history related to these infections; current diagnosis of substance dependence according to *Diagnostic and Statistical Manual of Mental Disorders* (DSM) fourth edition, text revision (American Psychiatric Association, 2000) criteria or a severe substance use disorder according to DSM-5 (American Psychiatric Association, 2013) criteria (except for nicotine or caffeine); current or past treatment (within two years) for a substance-related disorder; and positive urine drug or alcohol screen at admission to Qualification or Test Phases, except for tetrahydrocannabinolic acid (THCA) or benzodiazepines, which could be allowed at the investigators’ discretion due to the long time periods that these drugs can be detected in biological matrices.

### Endpoints

#### Drug effect using a VAS

Ratings of drug effects using a VAS were captured electronically using Cambridge Cognition software 2014 (Cambridge Cognition Ltd, Cambridge, UK). The primary endpoint was peak rating (*E*_max_) for Liking at the Moment across the first 12 h after drug administration using a self-reported 100-point bipolar liking/disliking VAS. *E*_max_ (and *E*_min_) values were calculated from the individual maximum (and minimum) values for each measure and for each participant. Key secondary endpoints were retrospective VAS ratings at 24 h after drug administration for Overall Drug Liking and how much the participant would like to Take the Drug Again (0 = not at all, 100 = very much). Other secondary endpoints, assessed using VAS, were: Disliking at the Moment (*E*_min_ on liking/disliking scale); Strength of Drug Effect (0 = no drug effect at all, 100 = very strong drug effect); positive subjective drug effects of Good Effects and High (both on scales 0 = “definitely not,” 100 = “definitely so”); negative subjective drug effects of Bad Effects and Anxiety (0 = “definitely not,” 100 = “definitely so”); and drug identification using Drug Similarity VAS (0 = “not at all similar,” 100 = “very similar”) at 2 h (momentary rating) and 24 h (retrospective rating) for similarity with 11 drugs or drug classes (opioid or pain killer, muscle relaxant, sedative/hypnotic, hallucinogen, stimulant, alcohol, nicotine, marijuana, phencyclidine, ephedrine, and caffeine). Alertness/Drowsiness, Agitation/Relaxation, Colors Brighter, and Sounds Louder were also assessed as secondary endpoints using a bipolar VAS with 50 as “neutral” on a scale of 0 to 100.

#### Addiction research center inventory

**Figure 1.**
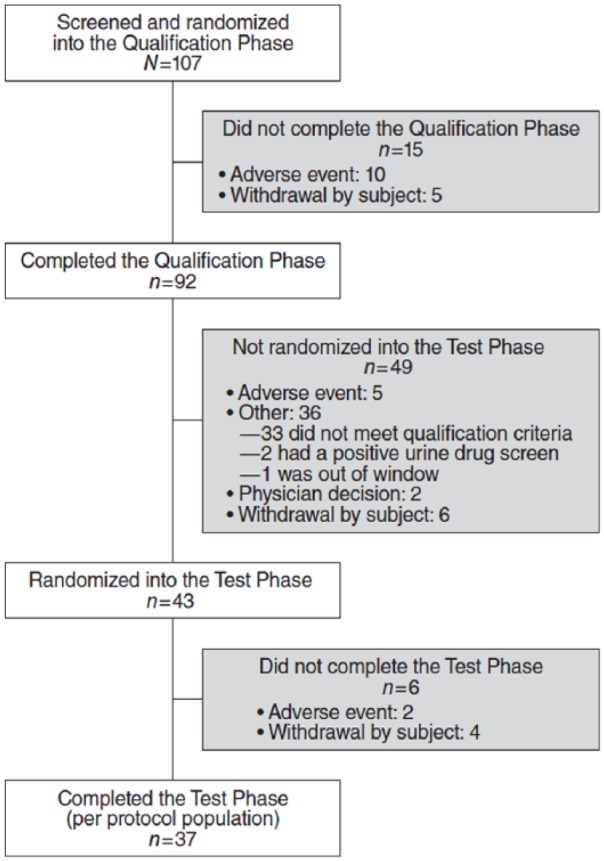
Disposition of participants.

The 49-item short form of the Addiction Research Center Inventory (ARCI; (Haertzen, 1965) was administered at 2 and 6 h after dosing as a secondary endpoint. The ARCI includes Amphetamine (A), Morphine-Benzedrine Group (MBG), Lysergic Acid Diethylamide (LSD), Benzedrine Group, and Pentobarbital Chlorpromazine Alcohol Group scales. The MBG, LSD, and A scales were of greatest interest because they are indicative of euphoric, dysphoric, and amphetamine-like effects, respectively. The 2-h data are presented for all ARCI scales, since this time point is closest to the peak subjective effects that was observed for each drug (Figure 1).

#### Subjective Drug Value

The assessment of Subjective Drug Value is a validated measure of abuse potential based on the amount of money that participants would pay to receive the drug again (Griffiths et al., 1996). It uses a series of independent, theoretical forced choices whereby participants express preferences to either receive a previously administered dose of drug or placebo, or an amount of money. The minimum and maximum values using this procedure were US$0.26 and US$47.97, respectively (Parasrampuria et al., 2007; Schoedel et al., 2010). Subjective Drug Value was assessed approximately 24 h after dosing.

#### Safety

Safety was evaluated for all treatments based on treatment-emergent adverse events (TEAEs), whether observed by the investigator, reported by the participant, or determined from laboratory findings. Additionally, vital signs were assessed at specified time points after dosing.

#### Statistical analysis

The study sample size was determined by a power analysis based on the differences observed between placebo and 45 or 90 mg of phentermine on the primary endpoint of Liking at the Moment from a previous study (Schoedel et al., 2012); these findings indicated that a sample size of 30 participants would have >95% power to detect significant differences at the (two-tailed) 5% level. Analyses of primary and secondary endpoints were performed on the per protocol population defined as participants who completed all six Test Phase treatments. Statistical significance for all non-parametric data (*E*_max_, *E*_min_, Overall Drug Liking, and Bad Effects) was evaluated using the Wilcoxon Sign Rank test. A mixed-model analysis of covariance was used for all parametric data. The model included treatment, period, and treatment sequence as fixed effects, baseline (pre-dose) measurement as a covariate where applicable, and participant nested within sequence as a random effect. For each endpoint, planned comparisons were conducted without multiplicity adjustment. All comparisons were two-tailed at the 5% significance level.

Because there were significant ratings of disliking after the 1200 mg dose of solriamfetol, a post hoc regression analysis was performed to explore the relationship between Bad Effects and Disliking at the Moment for the high doses of solriamfetol and phentermine. This regression evaluated *E*_min_ ratings of momentary disliking versus *E*_max_ ratings of Bad Effects.

All analyses were performed using SAS version 9.4 (SAS Institute, Cary, NC, USA).

## Results

### Participants

Study enrollment was initiated on 4 August 2014, and treatment concluded (last participant completed) on 13 November 2014. Of 107 participants who were screened and randomized to the Qualification Phase, 92 completed this phase and 43 were randomized to the Test Phase; see Figure 1. Among the randomized participants, none were positive for benzodiazepines at initiation of the Test Phase, and 22 (51.2%) were positive for cannabinoids (THCA) at this time point. The 43 enrolled participants were 74.4% male, 67.4% African American, and 32.6% White, with a mean (standard deviation (SD)) age of 29.3 (7.1) years (Table 1). Consistent with the inclusion criteria, all participants reported a history of recreational drug use from ⩾2 illicit drug classes including use of cocaine, amphetamine, methamphetamine, methylphenidate, or phentermine at least 10 times in the past five years and at least once in the past three months. Of these participants, 37 completed the study (Figure 1); four discontinued for personal reasons and two for TEAEs after having received solriamfetol 1200 mg. Thirty-four of the 37 participants (92%) in the per protocol population reported smoking cigarettes. Smoking behavior was not recorded during the study unless a participant smoked during times when smoking was prohibited (a protocol violation); no deviations pertaining to smoking were recorded.

**Table 1.** Baseline demographic and clinical characteristics (safety population).

Variable	Overall *N* = 43
Age, mean ± (SD), years	29.3 (7.1)
Sex, *n* (%)	
Male	32 (74.4)
Female	11 (25.6)
Race, *n* (%)	
White	14 (32.6)
Black or African American	29 (67.4)
Other	0
Weight, mean (SD), kg	76.8 (14.2)
Height, mean (SD), cm	172.9 (8.7)
BMI, mean (SD), kg/m^2^	25.6 (3.7)
Medical history, *n* (%)	
Headache	6 (14.0)
Acne	3 (7.0)
Gunshot wound	3 (7.0)
Female sterilization^a^	4 (36.4)

a *n*=11.

BMI: body mass index; SD: standard deviation

### Primary and key secondary endpoints

**Figure 2.**
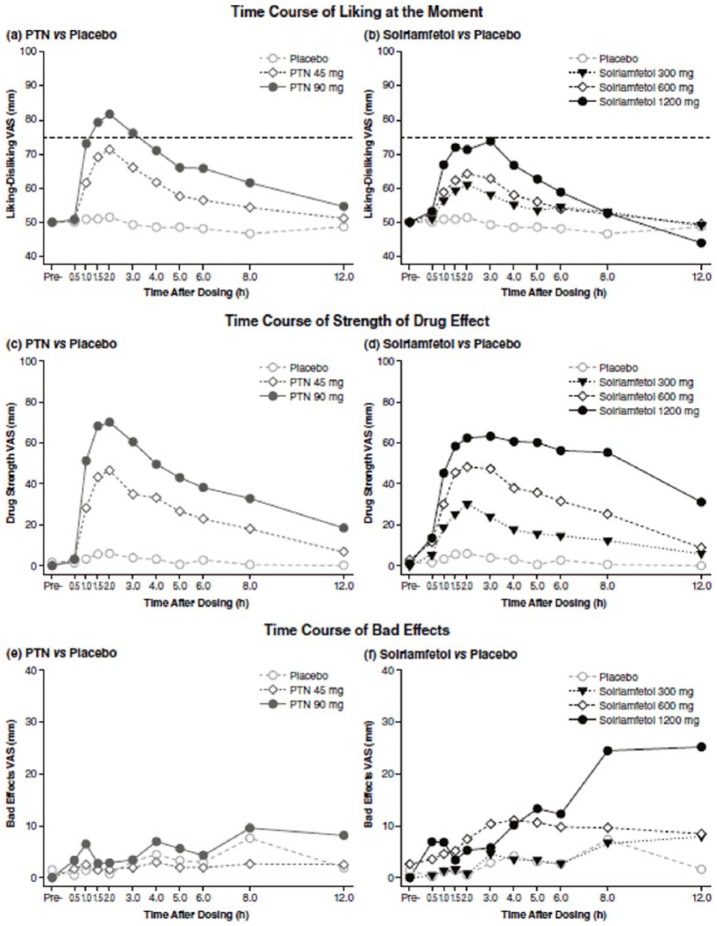
Mean ratings of (a) and (b) Liking at the Moment (dashed lines are for comparison of effect at maximum doses), (c) and (d) Strength of Drug Effect, and (e) and (f) Bad Effects over the first 12 h after dosing (*n =* 37). PTN: phentermine; VAS: visual analog scale

At doses that were found in this study to produce similar peak ratings of Strength of Drug Effect (e.g. phentermine 90 mg and solriamfetol 1200 mg or phentermine 45 mg and solriamfetol 600 mg), mean Liking at the Moment ratings for phentermine were numerically higher over the first 12 h of dosing, indicating greater liking of supratherapeutic doses of phentermine relative to supratherapeutic doses of solriamfetol; see Figure 2. The maximum mean liking effect for the high dose of phentermine 90 mg was larger and occurred sooner after drug administration than the peak for solriamfetol 1200 mg. A post hoc analysis of time to Peak Liking at the Moment (*E*_max_) for individual participants showed that the time of peak liking (*T*_max_) tended to be later after administration of solriamfetol 1200 mg than after phentermine 90 mg (156.9 (standard error (SE) 13.8) *vs.* 131.9 (13.1) min, respectively; *p*=0.09).

**Figure 3.**
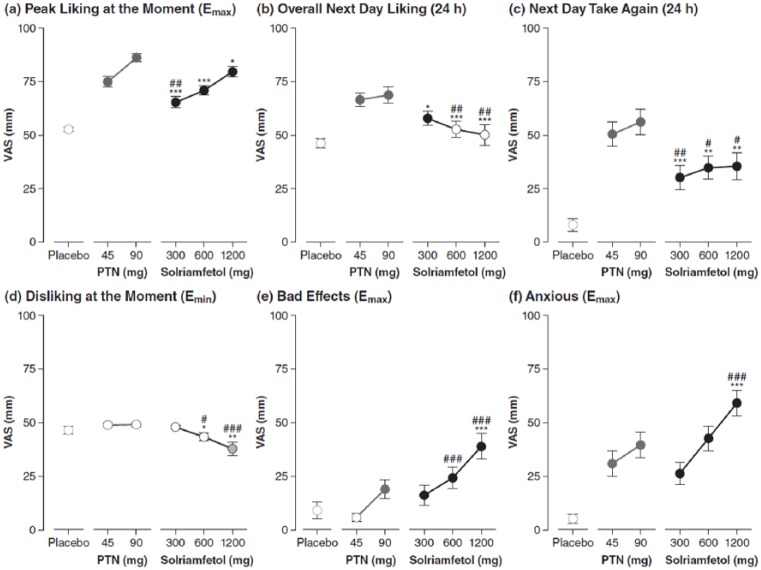
Perceptions of (a)–(c) positive and (d)–(f) negative drug effects. (a)–(c) Filled circles indicate significant differences versus placebo (*p*<0.05). The asterisk (*) and number (#) symbols indicate significant differences versus PTN 90 mg and 45 mg, respectively, with one, two, and three symbols corresponding to *p*<0.05, *p*<0.01, and *p*<0.001, respectively. Panels (a) and (b) are on 0–100 bipolar scales with 50 representing a neutral response. (d)–(f) Filled circles indicate significant differences versus placebo (*p*<0.05 solid fill; *p*=0.05 for shaded fill). The asterisk (*) and number (#) symbols indicate significant differences versus PTN 90 mg and 45 mg, respectively, with one, two, and three symbols corresponding to *p*<0.05, *p*<0.01, and *p*<0.001, respectively. Data are mean ± standard error. *E*_max_: peak effect; *E*_min_: lowest effect; PTN: phentermine; VAS: visual analog scale

All doses of solriamfetol and phentermine resulted in significantly higher ratings on the primary endpoint of Peak Liking at the Moment (*E*_max_) compared with placebo (*p*<0.001), which can be seen in Figure 3(a) and Supplementary Material Table S1 online. Peak Liking at the Moment for all doses of solriamfetol was significantly lower than phentermine 90 mg (*p*<0.001 for solriamfetol 300 and 600 mg; *p*=0.031 for solriamfetol 1200 mg). Figure 3(a) also shows that Peak Liking at the Moment for solriamfetol 300 mg was significantly lower than phentermine 45 mg (*p*=0.005); solriamfetol doses of 600 and 1200 mg were not different from phentermine 45 mg. On the next day evaluation of Overall Drug Liking, both doses of phentermine were rated as statistically higher than placebo (*p*<0.001), as was solriamfetol 300 mg (*p*=0.021); see Figure 3(b) and Supplementary Table S1. However, ratings of Overall Drug Liking for solriamfetol 600 and 1200 mg were not significantly different from placebo and were significantly less than both doses of phentermine (*p*<0.05). Overall Drug Liking for solriamfetol 300 mg was not significantly different than phentermine 45 mg, as shown in Figure 3(b). On next day ratings of how much the participants would like to Take the Drug Again, ratings were significantly greater for all doses of solriamfetol and phentermine relative to placebo. However, for all doses of solriamfetol, participants were significantly less willing to Take the Drug Again compared with either dose of phentermine (*p*<0.05); see Figure 3(c) and Supplementary Table S1.

### Positive and negative subjective effects

On positive subjective effects, ratings of Good Effects and High were significantly higher than placebo for both doses of phentermine and all doses of solriamfetol (all *p*<0.001; Supplementary Table S1). All doses of solriamfetol were rated significantly lower than phentermine 90 mg for Good Effects (*p*<0.05). On ratings of High, solriamfetol doses of 300 and 600 mg were significantly lower than those for phentermine 90 mg (*p*<0.001), whereas solriamfetol 1200 mg was similar to phentermine 90 mg.

On negative subjective effects, ratings of Disliking at the Moment (*E*_min_ on the liking/disliking scale) were significantly greater for solriamfetol 1200 mg compared with placebo (*p*=0.05). In addition, there was significantly greater disliking for solriamfetol 600 mg and 1200 mg compared with both doses of phentermine (*p*<0.05); see Figure 3(d).

When participants were asked whether they were feeling any bad effects of the drug, ratings were significantly higher than placebo with phentermine 90 mg and with all doses of solriamfetol (300 mg and 600 mg, *p*<0.01; 1200 mg, *p*<0.001); see Figure 3(e) and Supplementary Table S1. Relative to phentermine, ratings of Bad Effects were significantly higher with solriamfetol 600 mg and 1200 mg than with phentermine 45 mg (both *p*<0.001), and significantly higher for solriamfetol 1200 mg compared with phentermine 90 mg (*p*<0.001). The time course of bad effects, which can be seen in Figure 2, revealed that ratings of Bad Effects were numerically greater than placebo across the entire time course for the supratherapeutic doses of 600 and 1200 mg solriamfetol and that the ratings of Bad Effects at the supratherapeutic doses of solriamfetol tended to increase throughout the day; see Figure 2.

Ratings of Anxious (Figure 3(f)) and Agitation (Supplementary Table S1) were also significantly higher for solriamfetol 1200 mg than phentermine 90 mg (*p*<0.001 versus both phentermine doses). Solriamfetol 1200 mg had similar effects to phentermine 90 mg on other subjective ratings including Alertness, Sounds Louder, and Colors Brighter (Supplementary Table S1).

To further evaluate the relationship between Bad Effects and Disliking at the highest doses of each drug, a post hoc regression analysis demonstrated a stronger relationship between Bad Effects and Disliking for solriamfetol 1200 mg than phentermine 90 mg, which had *R*^2^ values of 0.6215 and 0.2662, respectively (Supplementary Material Figure S1 online). There were also fewer ratings of disliking after 90 mg phentermine than after 1200 mg solriamfetol (Supplementary Figure S1).

Since ratings of Overall Drug Liking at 24 h for the two higher doses of solriamfetol (600 and 1200 mg) were not significantly different from placebo, a post hoc analysis was conducted in which the primary and key secondary endpoints were summarized for solriamfetol 1200 mg based on whether participants reported next day overall liking (rating >50; *n*=18) or disliking (rating ⩽50; *n*=19) on the Overall Drug Liking scale at 24 h. For approximately half of the participants who reported next day liking of solriamfetol 1200 mg, none of the other primary and key secondary measures were numerically higher for solriamfetol 1200 mg compared with phentermine 90 mg. For approximately half of the participants who did not report next day liking for solriamfetol 1200 mg, all of the other primary and key secondary measures were markedly lower for solriamfetol 1200 mg compared with phentermine 90 mg. For example, mean (SE) ratings for solriamfetol 1200 mg *vs*. phentermine 90 mg were, respectively, 75.3 (3.9) *vs*. 84.8 (3.0) for Drug Liking at the Moment (*E*_max_); 27.3 (4.9) *vs*. 62.7 (6.2) for Overall Drug Liking at 24 h; and 10.1 (5.8) *vs.* 42.1 (8.8) for Next Day Take Drug Again.

### Addiction research center inventory

Results for all ARCI scales are summarized in Supplementary Table S1, with details of mean scores and pairwise comparisons shown in Supplementary Table S2. For the most relevant ARCI scales (MBG and LSD) at the 2-h time point, both solriamfetol and phentermine had dose-dependent effects that were significantly greater than placebo (Supplementary Table S2). Scores on the MBG scale, which is interpreted as a measure of euphoria, were significantly lower (*p*<0.05) at all doses of solriamfetol than with phentermine 90 mg, while scores on the LSD scale, which is interpreted as a measure of dysphoric effects, were significantly greater (*p*<0.05) with solriamfetol 1200 mg than both doses of phentermine. Solriamfetol 300 and 600 mg also had significantly lower scores on the A scale compared with phentermine 90 mg (*p*<0.05; Supplementary Table S2).

### Drug similarity VASs

Across the Drug Similarity VASs (Supplementary Table S3), placebo was appropriately identified as placebo-like in momentary and retrospective ratings, and phentermine was appropriately identified as stimulant-like at both evaluated time points. Solriamfetol at the supratherapeutic doses (600 and 1200 mg) was rated as stimulant-like to a similar extent as phentermine. Ratings of similarity to caffeine for phentermine and solriamfetol were intermediate to the ratings for phentermine and solriamfetol to placebo and stimulants.

### Subjective drug value

Based on mean value in dollars, both doses of phentermine and all doses of solriamfetol were rated significantly more valuable than placebo (*p*<0.001). Phentermine 90 mg (US$13.15 (SD US$12.87)) was rated as significantly more valuable than solriamfetol 300 mg (US$6.50 (SD US$8.69); *p*<0.001) and numerically higher than solriamfetol 600 mg (US$10.74 (SD US$13.29)) or solriamfetol 1200 mg (US$10.83 (SD US$13.85); *p*=0.056). None of the comparisons between solriamfetol and phentermine 45 mg (US$9.80 (SD US$13.18)) were significant.

### Safety

The overall incidence of TEAEs was dose dependent for solriamfetol and phentermine (Table 2). No serious or severe TEAEs were reported with any of the treatments, and there were two discontinuations due to TEAEs, nervousness and increased blood pressure (BP); both events occurred after solriamfetol 1200 mg at approximately one-half hour and 4 h, respectively, after dosing. These events were mild in severity and resolved without treatment within 24 h for the nervousness and three days for the increase in BP. The most common TEAE across treatments was hypervigilance; other common TEAEs ⩾ 10% with solriamfetol included elevated mood, dry mouth, hyperhidrosis, nausea, headache, decreased appetite, feeling of relaxation, restlessness, palpitations, paresthesia, and insomnia (Table 2). For hypervigilance, elevated mood, and feelings of relaxation, incidence rates were similar between solriamfetol 1200 mg and phentermine 90 mg.

**Table 2.** Treatment-emergent adverse events reported in the Test Phase among the safety population (*N* = 43).

TEAE	Number (%) of participants					
	Placebo	Solriamfetol			Phentermine	
	*n*=41	300 mg *n*=38	600 mg *n*=41	1200 mg *n*=42	45 mg *n*=40	90 mg *n*=40
Any TEAE	18 (43.9)	24 (63.2)	32 (78.0)	40 (95.2)	31 (77.5)	40 (100)
Discontinuations due to TEAEs	0	0	0	2 (4.8)	0	0
Serious TEAEs	0	0	0	0	0	0
Severe TEAEs	0	0	0	0	0	0
Most common TEAEs, ⩾ 10% of any treatment group						
Hypervigilance	4 (9.8)	14 (36.8)	12 (29.3)	18 (42.9)	16 (40.0)	18 (45.0)
Elevated mood	1 (2.4)	3 (7.9)	4 (9.8)	10 (23.8)	4 (10.0)	7 (17.5)
Dry mouth	1 (2.4)	2 (5.3)	5 (12.2)	9 (21.4)	4 (10.0)	9 (22.5)
Nausea	1 (2.4)	0	4 (9.8)	9 (21.4)	0	1 (2.5)
Feeling of relaxation	2 (4.9)	2 (5.3)	5 (12.2)	8 (19.0)	6 (15.0)	8 (20.0)
Decreased appetite	0	2 (5.3)	2 (4.9)	8 (19.0)	3 (7.5)	4 (10.0)
Hyperhidrosis	0	2 (5.3)	5 (12.2)	8 (19.0)	4 (10.0)	5 (12.5)
Insomnia	0	1 (2.6)	2 (4.9)	7 (16.7)	2 (5.0)	7 (17.5)
Headache	2 (4.9)	2 (5.3)	2 (4.9)	6 (14.3)	2 (5.0)	1 (2.5)
Restlessness	0	0	0	6 (14.3)	1 (2.5)	1 (2.5)
Palpitations	0	1 (2.6)	1 (2.4)	5 (11.9)	1 (2.5)	3 (7.5)
Paresthesia	0	2 (5.3)	5 (12.2)	3 (7.1)	3 (7.5)	6 (15.0)
Blood pressure increased	0	0	0	1 (2.4)	0	7 (17.5)
Irritability	1 (2.4)	0	2 (4.9)	0	4 (10.0)	3 (7.5)

TEAE: treatment-emergent adverse event

**Figure 4.**
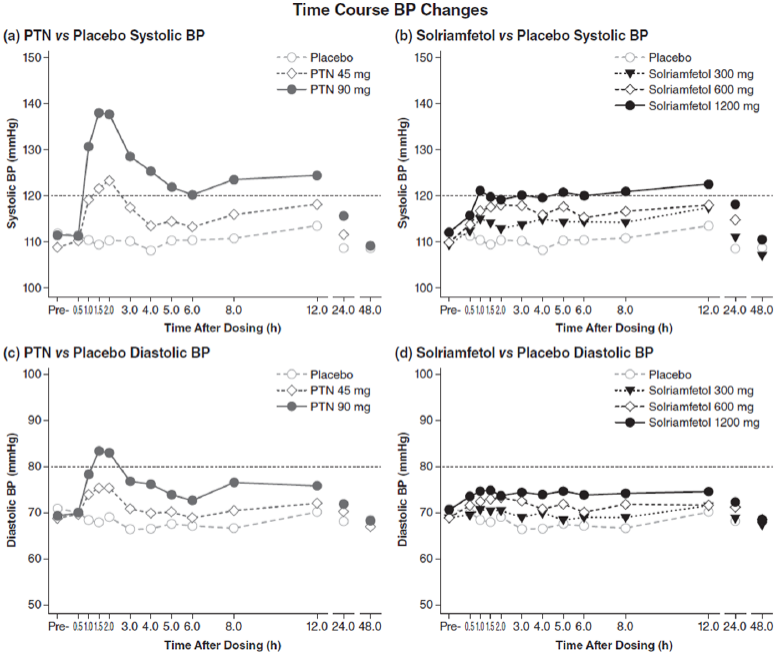
Time course of blood pressure changes after dosing. Dashed lines indicate upper limits normal for systolic and diastolic pressure. BP: blood pressure; PTN: phentermine

Although all the active treatment conditions were associated with some elevation of BP, both systolic and diastolic values were highest with phentermine (Figure 4). Over the first 6 h, the largest mean (SE) change from baseline in systolic BP was at 1.5 h after dosing with phentermine 90 mg, 26.6 (2.2) mmHg; see Figure 4(a). In contrast, changes in systolic BP, shown in Figure 4(b), were smaller with solriamfetol, with minimal changes at 300 mg, and a peak change of 8.9 (2.0) mmHg occurring 1 h after dosing with solriamfetol 1200 mg. Figure 4(c) illustrates the first 6 h after dosing; the largest mean (SE) change in diastolic BP from baseline was with phentermine 90 mg at 1.5 h, 13.9 (0.9) mmHg. There were minimal changes with solriamfetol 300 mg and a peak change of 4.2 (1.1) mmHg at 1.5 h with solriamfetol 1200 mg, as shown in Figure 4(d). BP values returned to baseline by 24 h after dosing with solriamfetol 300 mg, and by 48 h for all other active doses. Heart rate (HR) increased from baseline with placebo and phentermine, with the greatest mean (SE) increases 12 h after dosing: placebo 8.2 (1.2), phentermine 45 mg 13.1 (1.4), and phentermine 90 mg 13.8 (1.7) beats/min. Solriamfetol was associated with a dose-dependent increase in HR, peaking at 12 h after dosing; mean (SE) changes from baseline were 13.2 (1.5), 14.3 (1.5), and 20.2 (2.3) beats/min at doses of 300, 600, and 1200 mg, respectively. Although HR was still elevated at 24 h with the high doses of phentermine and solriamfetol, it was comparable to baseline by 48 h.

## Discussion

The overall results from this HAL study in recreational polydrug users reveal that solriamfetol has abuse potential that may be similar to or lower than the Schedule IV stimulant phentermine, and that there are substantial differences in the abuse potential profile, especially at supratherapeutic doses of solriamfetol; positive drug effects with solriamfetol were consistently lower than phentermine, and negative effects were consistently higher, which is reflected in statistically significantly lower ratings of Next Day Take Again for all doses of solriamfetol compared with either dose of phentermine. These findings are highly relevant to the scientific and regulatory assessment of the relative abuse potential of solriamfetol and might also help guide clinical practice and use of solriamfetol as an approved medication.

Study validity, including the use of phentermine as a positive control, was demonstrated by the observation that both doses of phentermine were significantly higher than placebo peak (*E*_max_) ratings of Drug Liking at the Moment over 12 h. The magnitude of peak ratings of Strength of Drug Effect over the first 12 h of dosing for solriamfetol and phentermine showed that the choice of dose range for both drugs, especially the high doses of 1200 mg for solriamfetol and 90 mg for phentermine, was appropriately matched, supporting the validity of the comparison for abuse potential. The Strength of Drug Effect of solriamfetol appeared to last longer than one would predict from the half-life (*t*_1/2_, 5–6 h) (Zomorodi et al., 2017), and the phentermine time course of Strength of Drug Effect was shorter relative to its half-life (*t*_1/2_, 20 h) (VIVUS, 2014). However, the 2–3 h time to *E*_max_ for solriamfetol effects on Drug Liking at the Moment and Strength of Drug Effect correspond with the median time to maximum plasma concentration (*T*_max_, 2-3 h) (Zomorodi et al., 2017), and the magnitude and time course of the effects of phentermine were consistent with what has previously been reported at these doses (Schoedel et al., 2012).

Solriamfetol resulted in dose-dependent ratings of Drug Liking at the Moment that were significantly higher than placebo at each of the three doses tested, ranging from 300 mg (highest anticipated therapeutic dose) to 1200 mg (highest tested dose in early clinical development). However, the ratings for the supratherapeutic doses (600 and 1200 mg) of solriamfetol indicated that they were liked significantly less than phentermine 90 mg. These two solriamfetol doses (600 and 1200 mg) also resulted in significantly greater disliking (*E*_min_) than phentermine 90 mg. The differences between solriamfetol and phentermine were even more pronounced for the retrospective ratings at 24 h, with the two highest doses of solriamfetol rated as significantly lower than both doses of phentermine for both Next Day Liking and Take Drug Again; solriamfetol 600 mg and 1200 mg had no differences from placebo for Next Day Liking. Importantly, ratings for solriamfetol also suggested that participants would be less likely to Take Drug Again relative to both phentermine doses, consistent with the lower euphoric effects assessed on the ARCI MBG scale. Other positive effects of solriamfetol were generally consistent with drug liking results, which were similar to or lower than phentermine 90 mg. However, ratings of High and drug value were not significantly different between solriamfetol 1200 mg and phentermine 90 mg. This distinction between the different measures suggests that ratings of High and drug value might be more indicative of the strength of drug effect rather than positive effects that are liked or the overall assessment of positive and negative subjective effects.

Results of the secondary measures of negative drug effects may help explain the lower solriamfetol ratings on the next day measures of Overall Drug Liking and Take Drug Again. The significantly higher ratings of Bad Effects of solriamfetol 600 and 1200 mg, which increased throughout the day and were also paralleled by a dose-dependent increase in ratings of Anxiety, demonstrate that negative drug effects become more pronounced over time and at higher doses of solriamfetol, which likely dampen the ratings of Overall Drug Liking and Take Drug Again, especially for supratherapeutic doses of solriamfetol. Additionally, in the post hoc correlation analysis the strength of the relationship between ratings of Disliking and Bad Effects for solriamfetol 1200 mg indicates that this dose produces consistent negative subjective effects in experienced recreational stimulant users. The higher *R*^2^ value relative to phentermine provides further support that the greater disliking that occurred with solriamfetol is related to its bad effects at this dose. The practical implications of these results are that abuse-related dose escalation of solriamfetol is unlikely because of the unpleasant effects at supratherapeutic doses including greater dysphoric effects than phentermine on the ARCI LSD scale. It should also be noted that in the post hoc responder analysis, more than half of the participants did not express any liking for solriamfetol 1200 mg at 24 h, and these participants also had markedly lower ratings of Drug Liking at the Moment and next day ratings of Take Drug Again compared with phentermine 90 mg. Furthermore, even participants who did express overall liking for solriamfetol 1200 mg at 24 h still had numerically lower ratings of Drug Liking at the Moment and next day ratings of Take Drug Again compared with phentermine 90 mg.

There were no serious adverse events after administration with solriamfetol despite inclusion of supratherapeutic doses of 600 and 1200 mg, of which the latter is approximately four times the highest planned therapeutic dose and is the highest dose that has been studied in humans. The safety profile of solriamfetol in this study was consistent with that observed in previous studies (Bogan et al., 2015; Ruoff et al., 2016). However, the highest overall rate of TEAEs with solriamfetol was in the group that received 1200 mg (>95%), the mean increase in HR was >20 beats/min, and there were two study discontinuations, for nervousness and increased BP, neither of which was serious, that occurred with solriamfetol 1200 mg, indicating that this dose is likely the highest dose that would have been tolerated. The lowest dose of solriamfetol, 300 mg, is the highest of the planned therapeutic doses. With regard to BP, substantial differences were observed between phentermine and solriamfetol, with phentermine resulting in greater increases in both systolic and diastolic BP at supratherapeutic doses. These effects provide support that solriamfetol may have pharmacodynamic properties different from those of traditional stimulants. The most commonly reported TEAEs were consistent with the wake-promoting profile as well as with what has been reported in phase 2 clinical trials of solriamfetol for the treatment of narcolepsy (Bogan et al., 2015; Ruoff et al., 2016).

### Strengths and limitations

This study was consistent with FDA guidance for abuse potential assessment (US Food and Drug Administration and Center for Drug Evaluation and Research, 2017), including use of recommended scales. However, there are several limitations, including that African Americans were over-represented in the study population compared with National Survey on Drug Use and Health estimates of the demographics of stimulant users (Substance Abuse and Mental Health Services Administration, 2013), although this is not expected to affect the conclusions. This study was not powered to evaluate differences by subgroups such as race or sex, but there were no apparent trends by these demographic subgroups. Another limitation is that the two-day washout was shorter than five half-lives for phentermine, although data such as those in Figure 2 suggest that subjective effect ratings returned to baseline at pre-dose. Pharmacokinetic data were not collected in this study, which precludes the evaluation of any within-study pharmacokinetic–pharmacodynamic analyses. Finally, approximately half of the participants had a positive cannabinoid drug screen at check-in to the residential research unit; however, recent cannabinoid use did not appear to have affected the pre-dose baseline ratings.

## Conclusion

Solriamfetol may have abuse potential similar to or lower than phentermine. More than half of the stimulant-using participants in this study did not report next day Overall Drug Liking after supratherapeutic doses of solriamfetol, next day ratings of Take Again were significantly lower for all doses of solriamfetol compared with either dose of phentermine, and ratings of negative subjective effects increased at higher doses of solriamfetol with two participants discontinuing participation after receiving the solriamfetol 1200 mg dose, which suggests that the likelihood of dose escalation and abuse of high doses of solriamfetol is low. There were no serious adverse events after administration with solriamfetol despite inclusion of supratherapeutic doses of 600 and 1200 mg, and the safety profile of solriamfetol was consistent with previous studies.

## Supplemental Material

suppl._mat – Supplemental material for A randomized, double-blind, placebo-controlled, crossover study to evaluate the human abuse liability of solriamfetol, a selective dopamine and norepinephrine reuptake inhibitor Click here for additional data file. Supplemental material, suppl._mat for A randomized, double-blind, placebo-controlled, crossover study to evaluate the human abuse liability of solriamfetol, a selective dopamine and norepinephrine reuptake inhibitor by Lawrence P Carter, Jack E Henningfield, Y Grace Wang, Yuan Lu, Debra Kelsh, Bradley Vince and Edward Sellers in Journal of Psychopharmacology
