# Supplementary material for: A randomized, double-blind, placebo-controlled, crossover study to evaluate the human abuse liability of solriamfetol, a selective dopamine and norepinephrine reuptake inhibitor
Source: J Psychopharmacol. 2018 Oct 1;32(12):1351–61. doi: 10.1177/0269881118796814 (PMC6247449; doi:10.1177/0269881118796814)
Supplement: suppl._mat – Supplemental material for A randomized, double-blind, placebo-controlled, crossover study to evaluate the human abuse liability of solriamfetol, a selective dopamine and norepinephrine reuptake inhibitor [file suppl._mat.pdf]

Figure S1. Relationship between Bad Effects (E_{max}) and Disliking at the Moment (E_{min}) over first 12 h after dosing (n=37). E_{max}: peak effect; E_{min}: lowest effect; PTN: phentermine.

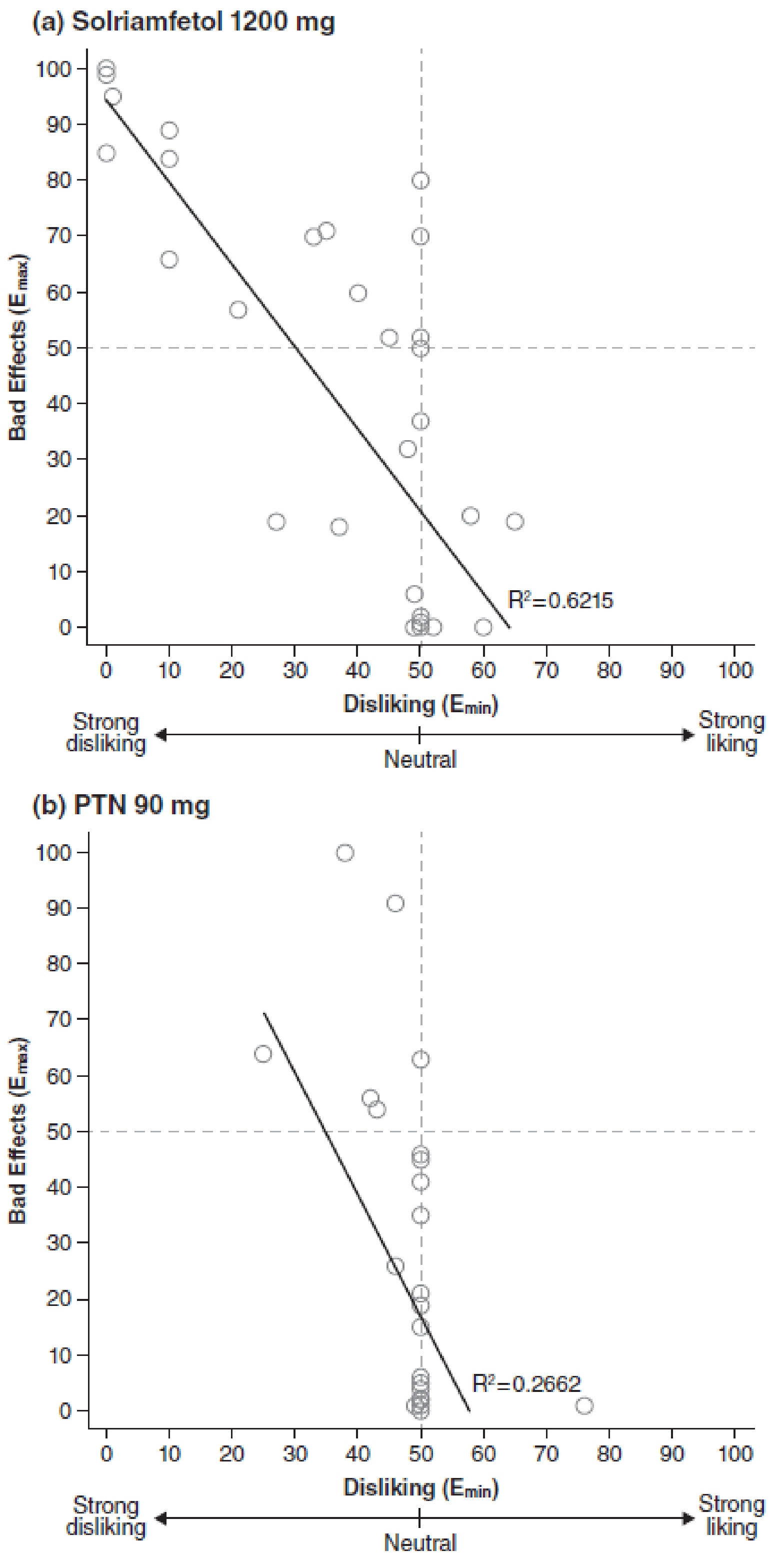

Table S1. Summary of pairwise comparisons of pharmacodynamics measures in the per protocol population (n=37).

**Measure**	**Phentermine *vs* Placebo**[1]	**Solriamfetol *vs* Placebo**[1]	**Solriamfetol 1200 mg vs Phentermine 90 mg**
**Positive Drug Effects**			
Drug Liking at the Moment E_{max}	+	+	Solriamfetol < phentermine
Overall Drug Liking at 24 h	+	+	Solriamfetol < phentermine
Take Drug Again at 24 h[2]	+	+	Solriamfetol < phentermine
Good Effects E_{max}	+	+	Solriamfetol < phentermine
High E_{max}	+	+	NS
Agitation/Relaxation E_{max}[2] (100 = very relaxed)	+	+	Solriamfetol < phentermine
ARCI MBG scale at 2 h[2]	+	+	Solriamfetol < phentermine
ARCI MBG scale at 6 h[2]	+	+	NS
Subjective Drug Value	+	+	NS
**Negative Drug Effects**			
Drug Disliking at the Moment E_{min}	NS	+	Solriamfetol > phentermine
Bad Effects E_{max}	+	+	Solriamfetol > phentermine
Anxious E_{max}[2]	+	+	Solriamfetol > phentermine
Agitation/Relaxation E_{min} (0 = very agitated)	+	+	Solriamfetol > phentermine
ARCI LSD scale at 2 h	+	+	Solriamfetol > phentermine
ARCI LSD scale at 6 h	+	+	Solriamfetol > phentermine

**Measure**	**Phentermine *vs* Placebo**[1]	**Solriamfetol *vs* Placebo**[1]	**Solriamfetol 1200 mg vs Phentermine 90 mg**
**Other Drug Effects**			
Strength E_{max}	+	+	NS
Detached E_{max}	+	+	NS
Alertness/Drowsiness E_{max}[2] (100 = very alert)	+	+	NS
Alertness/Drowsiness E_{min} (0 = very drowsy)	NS	NS	NS
Colors Brighter E_{max} (100 = brighter than usual)	+	+	NS
Sounds Louder E_{max} (100 = louder than usual)	+	+	NS
ARCI amphetamine scale at 2 h[2]	+	+	NS
ARCI amphetamine scale at 6 h[2]	+	+	NS
ARCI benzedrine scale at 2 h[2]	+	+	Solriamfetol < phentermine
ARCI benzedrine scale at 6 h[2]	+	+	Solriamfetol < phentermine
ARCI PCAG scale at 2 h	–	–	NS
ARCI PCAG scale at 6 h	NS	NS	Solriamfetol > phentermine

Note: Analysis was non-parametric unless otherwise specified. Measures for which the overall treatment effect was not statistically significant are not included in the table.

[1]"+"indicates that ratings for any dose were statistically higher than placebo, and "–" indicates that ratings for any dose were statistically lower than placebo.

[2]Parametric mixed-model analysis.

ARCI: Addiction Research Center Inventory; E_{max}: peak effect; E_{min}: lowest effect; LSD: Lysergic Acid Diethylamide; MBG: Morphine-Benzedrine Group; NS: not statistically significant; PCAG: Pentobarbital Chlorpromazine Alcohol Group.

Table S2. Mean scores on the ARCI scales at the 2-h time point (*n*=37).

**ARCI Scale**	**Mean Score (Standard Error)**					
	**Placebo**	**Solriamfetol 300 mg**	**Solriamfetol 600 mg**	**Solriamfetol 1200 mg**	**Phentermine 45 mg**	**Phentermine 90 mg**
MBG	2.8 (0.6)	6.1 (0.8)*‡	7.2 (0.8)*‡	8.2 (0.8)*§	7.0 (0.8)*	10.2 (0.7)*
LSD	2.7 (0.2)	4.2 (0.3)*§	5.4 (0.5)*§	5.9 (0.5)*§‖	3.6 (0.3)†	4.9 (0.4)*
Amphetamine	2.6 (0.4)	4.3 (0.5)*‡	5.3 (0.4)*‡	5.9 (0.5)*	5.1 (0.5)*	6.8 (0.4)*
Benzedrine	5.7 (0.3)	6.6 (0.4)‡	7.4 (0.5)†§	7.6 (0.5)†§	7.4 (0.4)*	8.8 (0.5)†
PCAG	4.2 (0.5)	3.1 (0.4)§	3.4 (0.5)	2.8 (0.5)*	3.8 (0.5)	2.2 (0.5)*

*$p<0.001$ and †$p<0.05$ vs placebo.

‡$p<0.001$ and §$p<0.05$ vs phentermine 90 mg.

‖$p<0.001$ vs phentermine 45 mg.

ARCI: Addiction Research Center Inventory; LSD: Lysergic Acid Diethylamide; MBG: Morphine-Benzedrine Group; PCAG: Pentobarbital Chlorpromazine Alcohol Group.

Table S3. Median drug similarity ratings on a visual analog scale (VAS; 0 = "not at all similar," 100 = "very similar") at 2 (momentary similarity) and 24 hours (retrospective similarity) after dosing (n = 37).

**Similarity comparison**	**Similarity VAS score, median (25%, 75% quartiles)**				
	**Phentermine**		**Solriamfetol**		
	**45 mg (n = 37)**	**90 mg (n = 37)**	**300 mg (n = 37)**	**600 mg (n = 37)**	**1200 mg (n = 37)**
Momentarily similarity (2 hours)					
Placebo	0 (0, 1)	0 (0, 0)	0 (0, 100)	0 (0, 0)	0 (0, 0)
Caffeine	5 (0, 52)	51 (12, 81)	7 (0, 39)	12 (0, 78)	29 (0, 77)
Stimulant	33 (0, 76)	92 (57, 98)	8, (0, 71)	63 (0, 89)	79 (39, 96)
Retrospective similarity (24 hours)					
Placebo	0 (0, 0)	0 (0, 0)	1 (0, 100)	0 (0, 0)	0 (0, 0)
Caffeine	4 (0, 49)	40 (6, 73)	1 (0, 71)	19 (1, 66)	29 (0, 66)
Stimulant	54 (0, 85)	91 (54, 100)	10 (0, 74)	63 (8, 96)	89 (71, 100)
